# Relationship between the optimal cut-off values of anthropometric indices for predicting metabolic syndrome and carotid intima-medial thickness in a Korean population

**DOI:** 10.1097/MD.0000000000017620

**Published:** 2019-10-18

**Authors:** Yu Jin Yang, Ho-Jong Park, Ki-Bum Won, Hyuk-Jae Chang, Gyung-Min Park, Yong-Giun Kim, Soe Hee Ann, Eun Ji Park, Shin-Jae Kim, Sang-Gon Lee

**Affiliations:** a Division of Cardiology, Ulsan University Hospital, University of Ulsan College of Medicine, Ulsan; b Division of Cardiology, Asan Medical Center, University of Ulsan College of Medicine, Seoul; c Division of Vascular Surgery, Ulsan University Hospital, University of Ulsan College of Medicine, Ulsan; d Division of Cardiology, Yonsei Cardiovascular Center, Yonsei University College of Medicine, Seoul; e Medical Information Center, Ulsan University Hospital, Ulsan, Republic of Korea.

**Keywords:** atherosclerosis, intima-medial thickness, metabolic syndrome, obesity

## Abstract

Anthropometric measurements are simple and useful methods for predicting metabolic syndrome (MetS) because obesity is a predominant feature of MetS. Although carotid intima-medial thickness (IMT) is generally used to evaluate subclinical atherosclerosis, the relationship between the optimal cut-off anthropometric values for predicting MetS and carotid IMT has not been analyzed in a Korean population.

Anthropometric measurements including waist circumference (WC), waist hip ratio (WHR), waist height ratio (WHtR), body mass index (BMI), and carotid IMT were assessed in 2560 Korean subjects without previous history of cardiovascular disease, cerebrovascular disease, neurological abnormalities, or malignancy who participated in baseline health examinations in a self-referral setting in the Seoul area between April 2010 and November 2012. MetS was defined using the National Cholesterol Education Program–Adult Treatment Panel III criteria.

In both men and women, the levels of all anthropometric indices were significantly higher in subjects with MetS than in those without MetS. According to the receiver operating characteristic curve, the values of 80.8 cm for WC, 0.87 for WHR, 0.52 for WHtR, and 24.6 kg/m^2^ for BMI were the optimal cut-offs for predicting MetS in women. The values of 89.3 cm for WC, 0.90 for WHR, 0.52 for WHtR, and 25.1 kg/m^2^ for BMI were the optimal cut-offs for predicting MetS in men. After adjusting for confounding factors, the WC optimal cut-off values for predicting MetS were independently associated with carotid IMT in both women and men (women: β = 0.016, *P* = .008; men: β = 0.033, *P* = .009). The optimal BMI cut-off value was independently associated with carotid IMT in men only (β = 0.027, *P* = .032).

Among anthropometric indices including WC, WHR, WHtR, and BMI, the WC optimal cut-off values for MetS were independently associated with an increased carotid IMT in both women and men in a Korean population.

## Abstract 2

Supplemental Digital Content is available in the text

## 1 Introduction

Metabolic syndrome (MetS) represents a cluster of several cardiovascular risk factors.^[1]^ It is certain that MetS is significantly associated with the development of atherosclerotic cardiovascular disease (CVD).^[2–4]^ The prevalence of MetS is rapidly increasing worldwide^[5,6]^; it affects approximately 31% of adults in Korea.^[7]^ Obesity is considered to have a pivotal role in the development of MetS.^[8,9]^ In clinical practice, several anthropometric indices, including waist circumference (WC), waist hip ratio (WHR), waist height ratio (WHtR), and body mass index (BMI), are used to evaluate obesity. The simple measurements of anthropometric indices are a useful tool for predicting the condition of MetS because obesity is a predominant characteristic of MetS. However, there has been considerable debate over which measurement is the most efficient in predicting metabolic risk factors,^[10–13]^ which could imply that different cut-off values for individual anthropometric index (segmented by sex and ethnicity) are needed to diagnose obesity with metabolic disorders. Especially, although WC is a major component to define MetS, it is important to identify the optimal cut-off of WC for the metabolically unhealthy obesity considering its close relationship with other metabolic disorders. In clinical practice, few data exist on the association between the optimal cut-off values of anthropometric indices for predicting MetS and subclinical atherosclerosis. In recent years, the assessment of subclinical atherosclerosis is generally performed using ultrasound to measure the intima-medial thickness (IMT) of the carotid artery. Therefore, the present study evaluated: optimal cut-offs of WC for the metabolically unhealthy obesity and those of other anthropometric indices for predicting MetS and the association between these values and carotid IMT after adjusting for confounding factors in a Korean population.

## 2 Methods

This is a cross-sectional investigation analyzing baseline data collected for a cohort study. We used data derived from 2560 subjects who participated in baseline health examinations in a self-referral setting in the Seoul area between April 2010 and November 2012. Subjects with a clinical history of CVD, cerebrovascular disease, neurological abnormalities, or malignancy were excluded, in accordance with the protocol of the present study. The study protocol was approved by our institution's ethics committee, and informed consent for the procedure was obtained from each individual.

All blood samples were obtained and analyzed after the subjects had fasted for at least 8 hours. The subjects wore light clothing and no shoes while their height, weight, and WC were measured. To ensure an accurate height measurement, subjects were asked to stand on a firm, level surface at a right angle to the vertical board of the measurement device. WC was measured at the midpoint between the lower border of the rib cage and the iliac crest. Hip circumference was measured around the widest portion of the buttocks. WHR was calculated as WC (cm)/hip circumference, while WHtR was calculated as WC (cm)/height (cm). BMI was calculated as weight (kg)/height (m^2^). MetS was defined as the presence of 3 or more of the following factors: abdominal obesity based on WC ≥ 90 cm in males or ≥80 cm in females; increased triglycerides ≥150 mg/dL; decreased high-density lipoprotein (HDL), defined as HDL cholesterol <40 mg/dL in males or <50 mg/dL in females; impaired fasting glucose, defined as fasting glucose ≥100 mg/dL, or established diabetes; and increased blood pressure including ≥130 mm Hg systolic or ≥85 mm Hg diastolic pressure, or on antihypertensive treatment, based on the National Cholesterol Education Program–Adult Treatment Panel III definition.^[1]^ Diabetes was established using one of the following indicators: fasting glucose ≥126 mg/dL, a referral diagnosis of diabetes, or an antidiabetic treatment.

Carotid IMT was measured using high-resolution B-mode ultrasonography (Acuson X300, Siemens Medical Solutions, Inc., Mountain View, California), with a transducer frequency of 13 to 15 MHz. Computer-assisted acquisition, processing, B-mode image storage, and the calculation of IMT were performed using the Syngo Arterial Health Package (Siemens). Automatic measurements of both common carotid arteries were made at the far wall of the 1 cm segment distal to the carotid bulbs. The mean value of both carotid IMT was used for analysis in the present study.

## 3 Statistical analysis

Values are expressed as mean ± SD for continuous variables and as numbers and percentages, n (%), for categorical variables. Continuous variables were compared using Student *t* test, and categorical variables were compared using the χ^2^ test or Fisher exact test, as appropriate. We carried out a receiver operating characteristic curve analysis using a Youden index to determine the optimal cut-off point of the individual anthropometric index, including WC, WHR, WHtR, and BMI, for predicting MetS in both men and women. Univariate and multivariate linear regression analyses were performed to evaluate the significance of the individual optimal cut-off of anthropometric indices for MetS in predicting subclinical atherosclerosis using carotid IMT. Multivariate models were adjusted for confounding factors, including age, smoking, diabetes, low-density lipoprotein (LDL), and other MetS components. SPSS version 18 (SPSS Inc., Chicago, IL) was used for all statistical analyses. Values of *P* < .05 were considered statistically significant.

## 4 Results

The baseline characteristics of the 2560 participants (60 ± 8 years, 33% men) are shown in Table 1. This study included 952 (37%) subjects with MetS and 1608 (63%) subjects without MetS. The mean values of all anthropometric indices and carotid IMT were significantly higher in women and men with Mets than in those without MetS (*P* < .001, respectively).

**Table 1:**
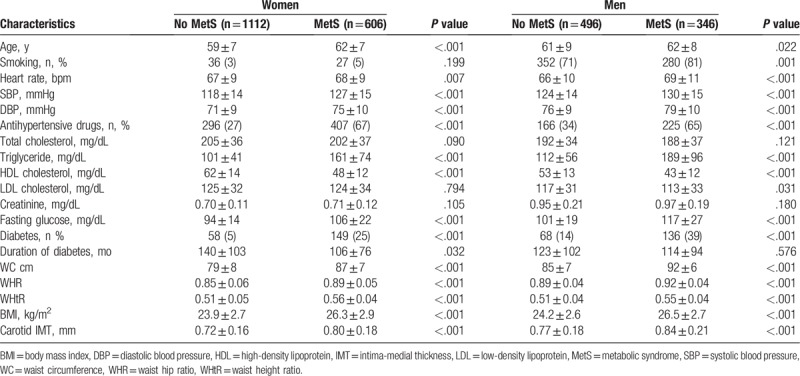
Baseline characteristics.

**Figure 1:**
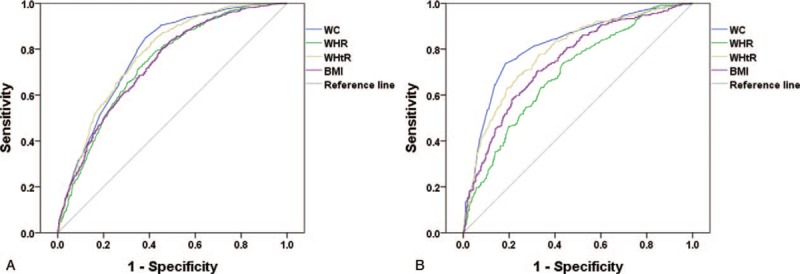
ROC curves for anthropometric indices to predict MetS in (A) females and (B) males. MetS = metabolic syndrome, ROC = receiver operating characteristic.

Table 2 and Figure 1 represent the gender specific area under the receiver operating characteristic curve and the optimal cut-off value of individual anthropometric indexes for predicting MetS. WC and WHtR yielded the highest AUC in women (WC: 0.78, 95% CI 0.76–0.80; WHtR: 0.78, 95% CI 0.75–0.80), and WC yielded the highest AUC in men (WC: 0.82, 95% CI 0.79–0.85). The values of 80.8 cm in WC, 0.87 in WHR, 0.52 in WHtR, and 24.6 kg/m^2^ in BMI were optimal for predicting MetS in women. The values of 89.3 cm in WC, 0.90 in WHR, 0.52 in WHtR, and 25.1 kg/m^2^ in BMI were optimal for predicting MetS in men.

**Table 2:**
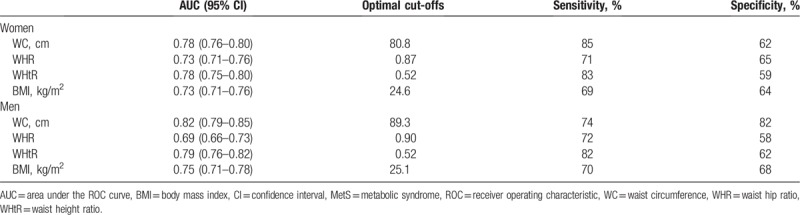
Area under ROC curve of different anthropometric indices in predicting MetS.

A univariate regression analysis for identifying the significance of optimal anthropometric cut-off values for MetS in predicting subclinical atherosclerosis revealed that the optimal cut-off values of WC (women: β = 0.058, *P* < .001; men: β = 0.053, *P* < .001), WHR (women: β = 0.041, *P* < .001; men: β = 0.043, *P* = .001), WHtR (women: β = 0.062, *P* < .001; men: β = 0.053, *P* < .001), and BMI (women: β = 0.027, *P* = .001; men: β = 0.028, *P* = .034) were significantly associated with carotid IMT. The multivariate regression analysis, after adjusting for age, smoking, diabetes, LDL, and other MetS components including increased blood pressure, increased triglycerides, decreased HDL, and increased fasting glucose, revealed that the optimal cut-off value of WC (women: β = 0.016, *P* = .037; men: β = 0.033, *P* = .009) was independently associated with carotid IMT in both women and men. The optimal cut-off value of BMI was independently associated with carotid IMT in men only (β = 0.027, *P* = .032) (Table 3).

**Table 3:**
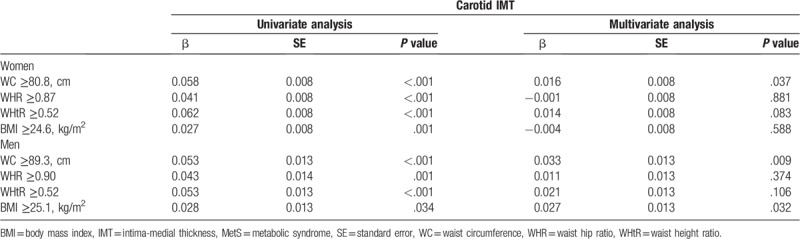
Relationship of optimal anthropometric cut-offs for predicting MetS to carotid IMT.

## 5 Discussion

To the best of our knowledge, this is the first study to evaluate: optimal cut-offs of WC for metabolically unhealthy obesity and those of other anthropometric indices for predicting MetS and the association between these values and carotid IMT in a Korean population. Among those anthropometric indices, that of WC was independently associated with an increased carotid IMT in both women and men.

Previously, several studies evaluated which anthropometric indices were the most efficient in predicting metabolic risk factors in a diverse ethnic population, but the results were inconsistent. Liu et al^[10]^ reported that WC, WHtR, and BMI were equally useful in predicting metabolic risk factors in the Chinese population. The San Antonio Heart Study^[11]^ reported that BMI and WC had a similar predictive usefulness for the development of MetS in non-Hispanic Whites and Mexican Americans. By contrast, the INTERHEART study reported that WHtR was a better predictor of metabolic risk factors (apart from hypertension) than other anthropometric indices, and that BMI alone was a better predictor of hypertension than other anthropometric indices.^[12]^ These inconsistent findings might be related to the different characteristics of obesity according to sex and ethnicity.

Obesity has been considered a major risk factor for the development of MetS because of its significant relationship with metabolic risk factors, such as glucose intolerance, dyslipidemia, and high blood pressure, which may influence adverse clinical outcomes related to CVD.^[14–17]^ In particular, the International Diabetes Federation (IDF) suggests that central obesity is compulsory for the diagnosis of MetS.^[18]^ Lim et al reported that central obesity and dyslipidemia were major factors in increasing the prevalence of MetS in Koreans for the past 10 years.^[7]^ For this reason, the measurements provided by anthropometric indices may be a simple and effective tool for predicting MetS. Considering that MetS is a useful concept in the prevention of CVD in a healthy general population,^[19]^ it is important to identify the relationship between the optimal cut-off anthropometric indices for MetS and subclinical atherosclerosis. However, data on this issue have been limited, especially in Asian populations. In the present study, WC yielded the highest AUC in both men and women because WC was one of the major criteria for the diagnosis of MetS. However, it was interesting that only the optimal cut-off value of WC for metabolically unhealthy central obesity was associated with subclinical atherosclerosis assessed using carotid IMT in both women and men, after adjusting for confounding risk factors. Thus, the measurement of WC might be effective for predicting both the status of MetS and subclinical atherosclerosis among anthropometric indices in a Korean population.

This study has some limitations. First, the present study includes only a Korean population; in fact, it may be the only attempt to identify the association between anthropometric cut-off values for MetS and subclinical atherosclerosis in a Korean population. Second, the potential for selection bias might be present because of the self-referral setting in the present study. Third, the impact of MetS on the progression of subclinical atherosclerosis may differ across different age groups.^[20]^ In this case, it was not possible to carry out a sub-analysis of different age groups because none of the cohort study participants were very young (Supplementary Table 1). Thus, it might be difficult to apply the present results to young people. Lastly, we have not been able to eliminate the possible effects of underlying medications on subclinical atherosclerosis because of the observational design of this study. Further large prospective studies will be required to address these issues.

## 6 Conclusion

The measurement of anthropometric indices could be a simple and effective method of predicting MetS status. Among anthropometric indices, including WC, WHR, WHtR, and BMI, the optimal cut-off values of WC for predicting MetS were independently associated with an increased carotid IMT in both women and men in a Korean population.

## Author contributions

**Conceptualization:** Yu Jin Yang, Ho-Jong Park, Ki-Bum Won, Hyuk-Jae Chang.

**Data curation:** Hyuk-Jae Chang.

**Formal analysis:** Ki-Bum Won, Eun Ji Park.

**Investigation:** Ho-Jong Park, Gyung-Min Park, Yong-Giun Kim, Soe Hee Ann, Shin-Jae Kim, Sang-Gon Lee.

**Software:** Yu Jin Yang, Ho-Jong Park.

**Supervision:** Ki-Bum Won, Hyuk-Jae Chang.

## Supplementary Material

Supplemental Digital Content
