# Supplementary material for: Relationship between the optimal cut-off values of anthropometric indices for predicting metabolic syndrome and carotid intima-medial thickness in a Korean population
Source: Medicine (Baltimore). 2019 Oct 18;98(42):e17620. doi: 10.1097/MD.0000000000017620 (PMC6824692; doi:10.1097/MD.0000000000017620)
Supplement: Supplemental Digital Content [file medi-98-e17620-s001.doc]

Supplementary table 1. Distribution of participants according to age groups

| Age (yrs) |  |
| --- | --- |
| 20−39 | 15 (0.6) |
| 40−49 | 200 (7.8) |
| 50−59 | 933 (36.4) |
| 60−69 | 1116 (43.6) |
| 70−79 | 286 (11.2) |
| 80−89 | 10 (0.4) |

Data are expressed as n (%).
